# Exotic-Dominated Grasslands Show Signs of Recovery with Cattle Grazing and Fire

**DOI:** 10.1371/journal.pone.0165758

**Published:** 2016-11-07

**Authors:** John T. Delaney, Raymond A. Moranz, Diane M. Debinski, David M. Engle, James R. Miller

**Affiliations:** 1 Ecology, Evolution, and Organismal Biology, Iowa State University, Ames, Iowa, United States of America; 2 Natural Resource Ecology & Management, Oklahoma State University, Stillwater, Oklahoma, United States of America; 3 Natural Resources & Environmental Sciences, University of Illinois Urbana-Champaign, Urbana, Illinois, United States of America; University of Saskatchewan, CANADA

## Abstract

In grasslands, overgrazing by domestic livestock, fertilization, and introduction of exotic forage species leads to plant communities consisting of a mixture of native and exotic species. These degraded grasslands present a problem for land managers, farmers, and restoration ecologists concerned with improving biodiversity while continuing to use the land for livestock production. Here we assessed the response of butterfly and plant community composition to the use of fire and moderate grazing by domestic cattle on degraded grasslands dominated by exotic plants. We evaluated change by comparing experimental pastures to two reference sites that were grasslands dominated by native plants. We used two burning and grazing treatments: 1) patch-burn graze, a heterogeneously managed treatment, where one third of the pasture is burned each year and cattle have free access to the entire pasture, and 2) graze-and-burn, a homogenously managed treatment, where the entire pasture is grazed each year and burned in its entirety every three years. We tested for change in the butterfly and plant community composition over seven years using Bray-Curtis dissimilarity measures. Over the course of seven years, degraded pastures in both treatments became more similar to reference sites with respect to the butterfly and plant communities. Only two butterfly species and two plant functional guilds exhibited significant linear trends over time, with varying responses. Compositional changes in both the butterfly and plant communities indicate that the use of moderate grazing and fire may shift butterfly and plant communities of exotic-dominated grasslands to be more similar to reference tallgrass prairies over time.

## Introduction

Anthropogenic alterations to ecosystems can cause changes in assemblages of plant species, resulting in ecosystems composed of both exotic and native species that differ from ecosystems composed of primarily native species in number of ways, including biodiversity, phenology, photosynthetic mode, productivity, and ecosystem services [1–5]. These altered ecosystems present a problem for land managers, farmers, and restoration ecologists concerned with increasing biodiversity [6–8]. In some cases, restoration within exotic-dominated ecosystems may focus less on complete removal of exotic species and instead on enhancement of functional attributes of plants and resources for native species [9–11]. Using this approach, we ask whether the combined use of grazing and fire on an exotic-dominated grassland can result in a grassland that is more similar to a native-dominated tallgrass prairie remnant in terms of its butterfly species and plant functional guild composition.

We chose to monitor butterflies because they are among the most commonly studied invertebrates [12], and have become a key group for biodiversity monitoring [13–18]. The necessity of research on butterfly conservation and restoration is illustrated by recent declines in butterflies observed in Europe [19–22], as well as the Midwestern United States [23], and global declines have been noted recently for Lepidoptera and invertebrates in general [24]. Degraded grasslands (grasslands consisting of a mixture of native and exotic species) may be worth preserving and restoring as habitat for butterflies because, for many butterfly species, exotic plant species have the potential to fill the roles of native species in terms of both adult and larval resources [25–28].

Domestic cattle grazing is gaining popularity as a restoration tool in exotic-dominated grasslands [29], even in areas with no native ungulate species [30]. Although domestic grazing is often the causative agent of exotic plant species dominance [31], careful manipulation of timing and intensity of grazing can reduce exotic plant species dominance [32]. Reducing cover of exotic plant species via grazing [33–36] can allow rarer plant species a chance to recover [37]. Using grazing to reduce exotic plant species cover does not work in every instance [38], and results will vary depending on the specific grazing regime employed [33]. For grazing to be effective at reducing exotic species dominance and increasing diversity, a management regime needs to alter the palatability of target species via fire and selectivity of individual plant species via manipulation of animal distribution within the pasture [30].

Patch-burn grazing has been proposed as a method for altering both palatability and herbivore selectivity, and can reduce the dominance of exotic plants in grasslands [38–40]. Patch-burn grazing works by applying fire to only a portion of a pasture each year, which influences the cattle’s grazing patterns such that they concentrate their grazing efforts in the most recently burned patch. Using this approach, cattle may more readily graze exotic species following a fire when the new growth of the exotic species is more palatable than in patches that are not as recently burned [38]. By concentrating grazing within a smaller area (within a patch) for a shorter period of time (e.g., one season), selectivity for plant species is reduced, resulting in all plant species being defoliated more evenly [38–41]. Patch-burn grazing thus shifts grazing behavior from preferential selection of individual plant species to preferential selection of the patch [42].

In this study we examined seven years of plant and butterfly response to grazing and fire management within the tallgrass prairie ecosystem of North America using two different treatments: 1) patch-burn graze, where fire is applied heterogeneously to the site, which influences grazing patterns, and 2) graze-and-burn, where fire is applied homogenously. Because the pastures used in this experiment were prairie remnants [43], we expected that the addition of fire and grazing could provide conditions that would release some of the native plant species. We hypothesized that butterfly community composition on degraded pastures would become more similar to reference sites (native-dominated tallgrass prairie remnants) with the application of fire and moderate grazing treatments because 1) these processes were central in structuring and maintaining the tallgrass prairie ecosystem historically, and 2) both grazing and fire are required for maintaining diversity in many grassland systems. We also investigated a secondary hypothesis, that fire and grazing would further facilitate improvement of plant functional guild composition (i.e., degraded grasslands would become more similar in composition to reference communities), whether the species are composed of natives or exotics. We focused on plant functional guild composition in this study because 1) previous research has shown butterflies correlate better with composition of plant functional guilds than overall plant species composition at our study sites [44] and 2) manipulation of plant functional guild composition may be a more realistic objective for restoration of degraded grasslands, where many of the plant species are exotic. Finally, we hypothesized that the two different grazing treatments would differ in their responses and that the patch-burn grazing would show a more rapid trajectory towards the reference sites.

## Methods

Our study sites were located in the Grand River Grasslands, an area spanning over 28,000 ha in Ringgold County, Iowa, and Harrison County, Missouri, U.S.A. (Table 1). Due to the abundance of grassland still present on the landscape, the Grand River Grasslands has been identified as the best opportunity to restore a functioning tallgrass prairie system in the entire Central Tallgrass Prairie ecoregion [45]. Before settlement by Europeans, the area was primarily tallgrass prairie, whereas today the Grand River Grasslands consists predominantly of degraded grasslands dominated by exotic plant species [46]. However, because of a high proportion of remnant native vegetation, it represents a landscape with a high potential for restoration.

**Table 1:** Sites used in this study. Classification into native- and exotic-dominated was based on the proportion of native plant cover in 2007, the first year of the experiment. Reference sites were native-dominated tallgrass prairies that were not grazed in this study but burned in their entirety on a three year rotation. Reference sites were not treatments per se, rather they were used to compare how the two grazing treatments differed from remnant, native-dominated, tallgrass prairies. See methods for explanation of the two grazing treatment types. Stocking rate is in Animal Unit Months per hectare (AUM ha^-1^).

Site	Treatment	Native Plant Cover (%)	Classification	Area (ha)	Stocking Rate (AUM ha^-1^)						
					2007	2008	2009	2010	2011	2012	2013
Ringgold North	Reference Site	96	Native- dominated	15.4	-	-	-	-	-	-	-
Pawnee	Reference Site	88	Native- dominated	21.8	-	-	-	-	-	-	-
Gilleland	Graze-and-burn	17	Exotic- dominated	31.2	2.6	2.5	2.6	1.6	2.2	2.7	2.1
Lee Trail	Graze-and-burn	44	Exotic- dominated	34.0	2.6	2.6	2.6	1.2	2.5	3.8	4.0
Pyland West	Graze-and-burn	25	Exotic- dominated	17.8	2.6	2.6	2.7	1.2	1.7	2.0	1.9
Pyland North	Patch-burn graze	22	Exotic- dominated	25.3	2.4	2.6	2.6	1.1	1.8	2.8	2.6
Pyland South	Patch-burn graze	43	Exotic- dominated	22.7	2.3	2.8	2.8	1.1	1.2	1.6	1.5
Ringgold South	Patch-burn graze	38	Exotic- dominated	32.4	4.4	3.6	4.2	1.9	1.6	3.0	2.4

The sites used in this study were a subset of sites from a larger research project established in 2006 that compared burn-only, patch-burn grazed, and burn-and-grazed pastures [47]. All treatments included sites with and without a recent history of grazing, as well as both remnant and previously tilled acreage. Sites were therefore distributed among treatments nonrandomly, so that each treatment contained a range of land use histories [47]. These land use histories are discussed in detail in McGranahan et al. [48]. Sites were categorized as either native-dominated tallgrass prairie remnants (used as reference only) or degraded pastures based on proportion of native plant canopy cover in 2007 (Table 1). Reference sites (n = 2) had >88% canopy cover of native plants, and all degraded pastures (n = 6) had <44% canopy cover of native plants (Table 1). Exotic plant species dominate all of the degraded sites. All of the study sites used in this project were on state owned lands. Authority to use these sites in this study was provided by the Missouri Department of Conservation for Pawnee and the Iowa Department of Natural Resources for all remaining sites. No endangered or protected species were collected for this project.

Patch-burn graze sites were delineated into three patches with one of the patches burned each year, resulting in a three-year fire return interval for each patch. Graze-and-burn sites were also delineated into three patches, but sites were burned in their entirety every three years. For both treatments there were no physical boundaries between patches; cattle had free access to the entire pasture. The year prior to the beginning of the experiment (2006), the average stocking rate was 3.9 Animal Unit Months (AUM) ha^-1^ (SD = 0.8). Over the seven years of the study the average stocking rate was 2.4 AUM ha^-1^ (SD = 0.8). Following 2010, stocking rate was altered annually based on end-of-season forage estimates at each site from the previous year to better achieve heterogeneity in vegetation height among patches within the patch-burn graze treatment (Table 1) [49]. Reference sites were also split into three patches, and the entire site was burned every three years, but not grazed. For all sites within the study, burns were conducted in mid-late March. One patch of each of the patch-burn graze sites was burned each year beginning in 2007, the graze-and-burn sites were burned in 2009 and 2012, and one reference site (Ringgold North) was also burned in 2009 and 2012 while the other (Pawnee) was burned in 2008, 2010 and 2013.

Sites differed in grazing and burning history before our study began (Table 2). Prior to the experiment, all degraded sites appeared to have experienced extended periods of intense grazing, applications of chemical fertilizer (likely N-based), and seeding of exotic forage species in the past (M. Moe, personal communication). Further details on the land use history at these sites can be found in Debinski et al. [43] and McGranahan et al. [48]. We perceive the diversity of land-use histories as a benefit to our analysis in this study because this provides a greater representation of degraded grasslands on the landscape and because the difficulty of detecting trends in less controlled studies makes a significant result more robust.

**Table 2:** Recent land use history of cattle pastures. Recent history is up to five years prior to 2006.

Site	Treatment	Recent Grazing History	Recent Burn History
Gilleland	Graze-and-burn	At least 5 years rest	Not burned
Lee Trail	Graze-and-burn	At least 5 years rest	Last burned in 2004
Pyland West	Graze-and-burn	Heavily up to 2006	Not burned
Pyland North	Patch-burn graze	Heavily up to 2006	Not burned
Pyland South	Patch-burn graze	Heavily up to 2006	Not burned
Ringgold South	Patch-burn graze	At least 5 years rest	Last burned in 2003

### Sampling

Percent native cover estimates for the plant community came from two 500m^2^ permanently marked modified Whittaker plots [50] per patch (n = 6 Whittaker plots per site) sampled twice during the summer season [51]. Sampling occurred once in late May-early June and once in August. Locations of these plots were selected randomly within each of the two most dominant soil types in the study area. Canopy cover of each species was sampled from 10 0.5m^2^ subplots systematically placed within each Whittaker plot. Cover was estimated using the cover classes: 0, <1%, 1–5%, 6–25%, 25–50%, 51–75%, 76–95%, 96–100%. The canopy cover value for each species was the maximum canopy cover value observed. Further details on Whittaker plot placement and sampling can be found in McGranahan [51]. Information from the Whittaker plots was used only to categorize the sites based upon their percent native cover and not used in any of the analyses in this study.

Within each patch two permanent 100m butterfly transects were established at the beginning of the experiment. Thus, each site had a total of six transects. Starting points for each transect were 10m west of permanent Whittaker plots. The distance between transects varied for each patch because of the random placement of the Whittaker plots. Each transect was oriented from north to south and positioned within one patch for the entire length. Transects were sampled twice per year to include the two major butterfly emergence periods (mid-June and mid-July) using a modified “Pollard walk” method [52]. Observers walked the 100m transect at a steady pace (~10m/min) and recorded butterflies seen within a 5x5m area in front of the observer. Butterflies were identified to the species level on the wing where possible. Alternatively, they were captured and identified in the field or lab. If it was necessary to net a butterfly, the stopwatch was stopped so that handling time was not included in the sampling time. Surveys were conducted between 0930-1830h when the sun was not obscured by clouds, winds were less than 16km/hr, and temperatures were between 21–35°C [43].

Canopy cover of functional guilds was measured in mid-July using thirty 0.5-m^2^ (0.5x1.0m) Daubenmire [53] quadrats per patch arranged along three patch-wide transects, resulting in a total of 90 quadrats per site [47]. Variables measured included canopy cover of plant functional guilds: warm-season grasses (C4), cool-season grasses (C3), non-leguminous forbs, leguminous forbs, woody plants, and tall fescue. We separated tall fescue from other cool-season grasses because this species was the invasive grass species that was most abundant on our cattle pastures, and we were interested in tracking that separately. We estimated the canopy cover of each functional guild using the following cover class scale: 0, <1%, 1–5%, 6–25%, 26–50%, 51–75%, 76–95%, 96–100%. Compared to the species level information collected at a fine scale in the Whittaker plots, the Daubenmire estimates of functional guild cover sample a larger area and are therefore more representative of the composition of the entire pasture. Functional guild canopy cover is a useful predictor of vegetation composition for tallgrass prairie [54]. In 2007, quadrat data from one of the reference sites for two of the three patches at one site (Ringgold North) were lost, so we used the single patch in 2007 to represent the entire site for that year. We believe this to be an acceptable alternative since this site is particularly homogenous across patches.

### Statistical Analysis

To quantify change over time, Bray-Curtis dissimilarity between reference and grazed sites was calculated for the butterfly community and the plant groups. For each site, butterfly abundance was totaled among the three patches and percentage estimates for plant cover was averaged among patches. Comparisons between grazed sites and reference sites occurred only within each individual year; i.e. no comparisons in dissimilarity were made among years. Species abundances (for butterflies) and cover by functional guild (for plants) were averaged across the two reference sites for each year and dissimilarity of each grazed site during each year to the average of the reference sites was calculated using the vegdist function in the vegan package [55] in R version 2.12.1 [56]. Temporal trends in dissimilarity between experimental and reference sites were assessed for the butterfly and plant community (i.e. plant functional guilds) using a linear mixed-effects model with the lme function in the nlme package [57] in R. Fixed effects included year (2007–2013), treatment, stocking rate, precipitation, and a year by treatment interaction term. Treatment was a categorical variable, while all other fixed effects were treated as continuous variables. Each term was tested for a linear response to year, and for each model we tested whether a linear or quadratic term for year performed better. Site (categorical) was included in the models as a random effect to account for repeated measures over time. We initially tested all interaction and non-significant interactions were removed from the models (using Bonferroni correction thresholds), we retained the year*treatment interaction even though it was non-significant because it was an important component of our hypothesis that the two treatments would differ in their response.

Precipitation data were collected from National Oceanic and Atmospheric Administration’s National Climatic Data Center [58]. We extracted total growing season precipitation beginning on April 1^st^ and ending on the last butterfly sampling day of each year. Data were obtained from five weather stations within a 25-km radius of our study area and the average was used to represent an annual precipitation estimate for the study area.

We tested for changes in species abundance of 13 of the most abundant butterfly species and the six plant functional guilds. Our data showed high inter-annual variation in abundance of individual species and individual plant functional guilds (S1 and S2 Tables). Because of this variation, we calculated the difference in abundance of each butterfly species or canopy cover of each functional guild between each degraded site and reference site average within each year, in a similar manner as the calculation for compositional dissimilarity. We then tested for a linear relationship with year for each species or functional guild. The models were constructed identically to the model for compositional change. We used Bonferroni corrections to determine whether relationships were significant after multiple tests. The threshold p-value was 0.004 (α = 0.05/13) for the butterflies and 0.008 (α = 0.05/6) for plant functional guilds. All graphs in this manuscript were constructed using the ggplot2 package [59] in R.

## Results

**Fig 1:**
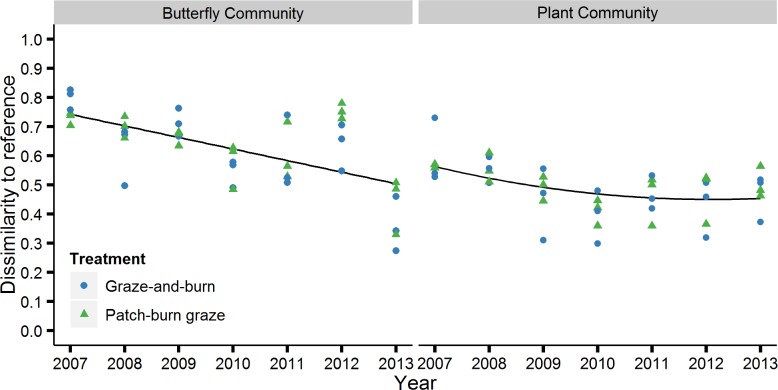
Convergence (i.e., declining dissimilarity) of butterfly community and plant community within degraded sites to reference sites over time. The dissimilarity measure on the y-axis is Bray-Curtis Dissimilarity, which was calculated as the dissimilarity between each grazed site and the average of the two reference sites for each year. See Table 3 for test of the response of decreasing dissimilarity over time.

Butterfly community composition became more similar to reference sites over the seven year period (Table 3; Fig 1). Composition of plant functional guilds also showed a significant relationship with time (Table 3; Fig 1). A quadratic relationship was determined to be a better fit for the plant response based upon the lowest AIC value and the delta AIC from this comparison was 10.24, indicating a strong difference between the two models [60]. The change in plant functional guild cover was especially strong in the first four years before leveling out in the final three years of the experiment. There were no significant differences between grazing treatments nor significant year by treatment interactions.

**Table 3:** Results of linear mixed-effects model analyses of change in dissimilarity of the butterfly community and plant community. Bray-Curtis dissimilarity was calculated between grazed sites and the average of reference sites within each year. Models included year (2007–2013), treatment (patch-burn graze and graze-and-burn), stocking rate of each site (AUM ha^-1^), and growing season precipitation as fixed effects. Table presents both the numerator (num) and denominator (den) degrees of freedom (DF). Stocking rate was included in the model because it changed each year at each site (Table 1).

	Fixed Effects	Value	Std.Error	numDF	denDF	t-value	p-value
Butterfly Community	Year	-0.06077	0.01082	1	32	-5.62	<0.001
	Treatment	-37.23752	30.46256	1	4	-1.22	0.289
	Stocking Rate	-0.01174	0.02025	1	32	-0.58	0.566
	Precipitation	-0.00004	0.00001	1	32	-3.34	0.002
	Year*Treatment	0.01854	0.01516	1	32	1.22	0.230
Plant Community	(Year)^2^	-0.33378	0.086017	1	32	-3.88	<0.001
	Treatment	0.01400	0.059351	1	4	0.24	0.825
	Stocking Rate	0.02737	0.014448	1	32	1.89	0.067
	Precipitation	-0.00001	0.00007	1	32	-1.66	0.106
	(Year)^2^*Treatment	0.17606	0.121791	1	32	1.45	0.158

**Fig 2:**
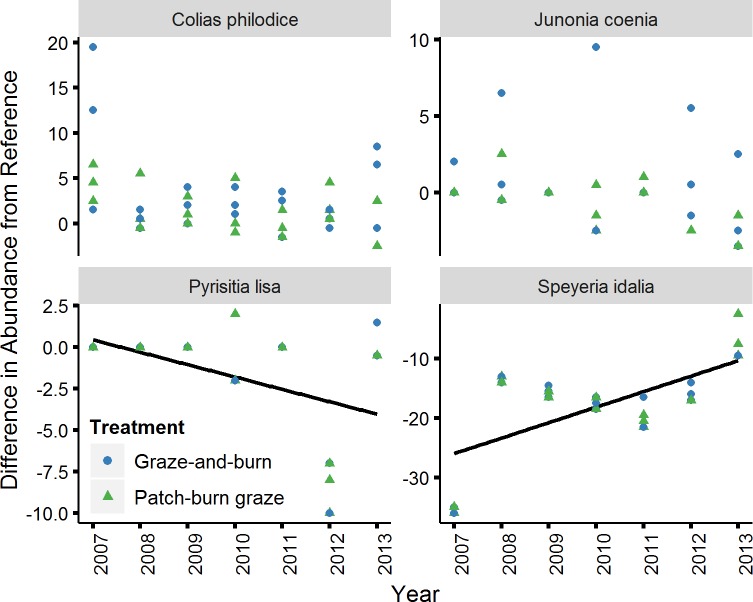
Difference in abundance from reference site of four butterfly species. Linear trends in species abundance at degraded sites were tested using the difference between the abundance at each degraded site and the average abundance of the two reference sites. All species presented in this figure showed a significant relationship with year below α = 0.05. Lines depict relationships that were significant when considering Bonferroni correction (p < 0.004). *Note*: *scale on the Y axis is different for each species*.

Four butterfly species showed a linear response with year below the α = 0.05 level, but only two of these relationships were statistically significant after accounting for multiple tests using a Bonferroni correction (p<0.004; Fig 2). *Pyrisitia lisa* (Little Yellow) and *Speyeria idalia* (Regal Fritillary), showed a significant linear trend (of increasing difference from reference for *Pyrisitia lisa* and decreasing difference from reference for *Speyeria idalia*) over the seven year period (Fig 2; df = 32; t = -3.41; p = 0.002; and df = 32; t = 8.50; p <0.001; respectively). However, these relationships appear to be more of an artifact of fluctuating inter-annual abundance patterns. *Pyrisitia lisa* butterflies were somewhat common in the last two years of the experiment and virtually absent in the first three years of the experiment, while *Speyeria idalia* showed a particularly high abundance at one reference site in 2007 and a particularly low abundance in 2013 at that same site, with little change during intermediate years (Fig 2). We removed *Pyrisitia lisa* and *Speyeria idalia* from our analyses of the butterfly community and reran the model testing for compositional change over time in the butterfly community. Their removal did not alter the interpretation of the previously presented butterfly community results.

**Fig 3:**
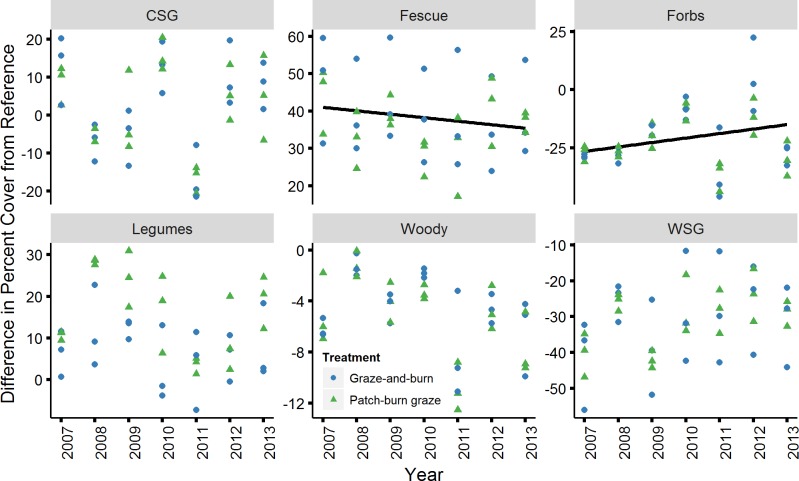
Difference in percent canopy cover of plant functional guilds from reference sites. Abbreviations are as follows, CSG = cool-season grasses (C3), Fescue = tall fescue (*Schedonorus arundinaceus* (Schreb.) Dumort., nom. cons.), Forbs = non-leguminous forbs, Legumes = leguminous forbs, Woody = woody vegetation, WSG = warm-season grasses (C4). Linear trends in functional guild cover at degraded sites were tested using the difference between the percent cover at each degraded site and the average percent cover of the two reference sites. Lines depict relationships that were significant when considering Bonferroni correction (p < 0.008). *Note*: *scale on the Y axis is different for each functional guild*.

For the plant functional guilds, both forbs and tall fescue exhibited a significant trend over time (Fig 3) after Bonferroni correction (p < 0.008). Warm-season grasses exhibited a relationship with time below α = 0.05 (i.e., p = 0.025), but not below the Bonferroni correction threshold. The difference between grazed sites and reference sites decreased over time in terms of forb cover (d.f. = 32; t = 3.96; p = 0.0004). The difference between grazed sites and reference sites decreased in terms of the cover of tall fescue (d.f. = 32, t = -3.35, p = 0.002; Fig 3), yet remained quite different from reference sites. Tall fescue was virtually absent from reference sites and using the raw data, the percent change in tall fescue cover was small for both treatments (patch-burn graze $\bar{x}$ = 2.7, SD = 0.20 and graze-and-burn $\bar{x}$ = 2.7, SD = 0.80). However, the average percent change in tall fescue was larger using the fitted estimates from the model that incorporated the variation contributed by changes in stocking rate and changes in precipitation (patch-burn graze $\bar{x}$ = 12.7, SD = 8.29 for and graze-and-burn $\bar{x}$ = 23.2, SD = 4.13). The average percent change in tall fescue did not differ significantly between the two treatments (df = 4, t = -1.97, p = 0.121). For both forbs and tall fescue, none of the other fixed effects were significant below the p = 0.008 threshold for multiple tests.

## Discussion

Degraded grasslands managed with grazing and fire showed signs of recovery as measured by increasing similarity to reference sites over seven years. Butterfly communities showed an approximately 25% increase in similarity to the reference sites over the seven year period. For the plant functional guilds, the change in similarity was less distinct, with an increase of ~15% in the first four years of the experiment but leveling off in the final three years. We did not find treatment-associated differences for the plant functional guilds or the butterflies. However, stocking rates were fairly similar between the two grazing treatments, and the number of grazing animals can have more of an effect on the response than the specific grazing treatments employed [61,62]. Although the degraded sites remained quite different from reference sites in the final year of the study, the degree of change observed here is encouraging considering the short time period and the minimal amount of management required (a three year fire return interval and moderate cattle grazing). That is not to say that increasing the fire return interval or stocking rate of cattle would produce quicker results, but rather the technique investigated here is less intensive compared to restoration techniques that focus on complete revegetation.

The dissimilarity between grazed sites and reference sites was lower for the plant community than the butterfly community throughout the study, which may be a function of separating the plant community into only six functional guilds. However, the composition of plant functional guilds became more similar to reference sites over the study period. Comparable changes in plant functional guild composition have resulted from grazing in other studies [63–66]. For example, Woodcock et al. [9] demonstrated a decrease in dissimilarity of plant functional characteristics between grazed and reference sites in Europe.

Stocking rate was adjusted annually and individually for each pasture as part of an adaptive management strategy to better achieve structural heterogeneity among patches in the patch-burn graze treatment [49]. Specifically, the stocking rate was highest in the first three years of the project (2007–2009) then was lowered in 2010 and then brought back up to an intermediate level in 2012 (Table 1). We accounted for this change in stocking rate in all of our models. Leveling off of the change in dissimilarity in the final three years of the study might be explained by the fact that stocking rate was lowered below a threshold where grazing could significantly affect change in the cover of plant functional guilds. For instance, Hickman et al. [61] found greater enhancement of plant diversity in high vs. moderate and low stocking densities. Tall fescue may have also created an alternative stable state making the grasslands resistant to change under the influence of grazing similar to its theoretical resistance to the influence of fire [67].

The positive response of butterflies to restorative grazing is consistent with results of other studies of butterfly responses to restoration through cattle grazing [68,69]. Dissimilarity between degraded and reference sites increased in 2012, most likely due to an extreme drought that may have differentially affected community composition of reference and degraded sites. Changes in weather patterns such as precipitation can translate to profound changes in individual butterfly species and community composition [70,71]. Degraded sites resumed their trajectory toward reference sites in 2013. Considering the variability in growing season precipitation and other variable weather patterns observed over the entire course of this experiment, it is encouraging that we were able to detect a significant change in butterfly composition.

The changes in composition of butterflies and plant functional guilds did not mirror each other to the degree that we had anticipated for this study. Previous research at our study sites indicates that composition of butterflies is correlated with the composition of plant functional guilds [44] and other studies have demonstrated similar relationships [72–77]. For example, Schaffers et al. [74] found that plant species composition was the best predictor of all seven of the arthropod taxa they studied compared to other commonly measured predictors. It is possible that butterflies are responding to changes other than plant functional guild cover that were not investigated here. Alternatively, there could be a lag between changes in the plant community and the responses of the butterflies, especially for those butterfly species with long generation times (e.g., one brood/year). A lag effect could explain why the butterflies continued on a convergent trajectory over all seven years while the plant community trajectory leveled off after four years. Researchers studying European grasslands also demonstrated a lag between restoration and the response of butterflies following revegetation [18].

In addition to the overall change in butterfly community composition, we were able to detect significant trends in two individual butterfly species. However, these two species were not drivers of the change observed in butterfly composition, because the removal of those species from the dataset did not change the interpretation of the results. These results highlight the utility of analyzing multivariate responses to environmental change. Organisms can fluctuate in abundance along temporal time scales due to biotic interactions and in response to environmental factors [78]. Weather, parasitoids, predators, and competition, influence individual butterfly species abundance such that fluctuations from year to year can be large [71]. By annually comparing degraded sites to reference sites, we reduce the noise in the relationships and buffer against erroneous conclusions about the importance of specific species.

### Implications for Management

The pervasiveness of exotic species in native ecosystems is a major problem for land managers throughout the globe, and emerging threats of increasing human population, agricultural intensification, climate change, and limited resources further complicate the issue [8]. In agricultural grasslands, many of the exotic plant species have reached a threshold where complete and permanent removal is unlikely without intensive, costly intervention. Many of these exotic species may be useful for conservation if they provide a resource for higher trophic levels [79]. For example, exotic plant species that produce nectar can be valuable resources for native butterflies [25–28]. This work demonstrates that management with moderate grazing and fire has potential to restore degraded grassland habitat and increase grassland butterfly biodiversity in the presence of exotic plant species dominance while providing an economic return in the form of cattle production. The approach presented here has great potential for success in extensive landscapes where intensive management of the entire area is not economically feasible.

## Supporting Information

S1 Table Average percent cover for each of the six functional groups at each site by year. Abbreviations are as follows, CSG = cool-season grasses (C3), Fescue = tall fescue (*Schedonorus arundinaceus* (Schreb.) Dumort., nom. cons.), Forbs = non-leguminous forbs, Legumes = leguminous forbs, Woody = woody vegetation, WSG = warm-season grasses (C4).
(DOCX)

S2 Table Total abundance of the 14 most common butterfly species for each site by year. Total abundance is the sum of all butterflies counted from six transects within each site in each year.
(DOCX)
