# Supplementary material for: Exotic-Dominated Grasslands Show Signs of Recovery with Cattle Grazing and Fire
Source: PLoS One. 2016 Nov 7;11(11):e0165758. doi: 10.1371/journal.pone.0165758 (PMC5098731; doi:10.1371/journal.pone.0165758)
Supplement: S1 Table — Abbreviations are as follows, CSG = cool-season grasses (C3), Fescue = tall fescue (Schedonorus arundinaceus (Schreb.) Dumort., nom. cons.), Forbs = non-leguminous forbs, Legumes = leguminous forbs, Woody = woody vegetation, WSG = warm-season grasses (C4). (DOCX) [file pone.0165758.s001.docx]

**S1 Table: Average percent cover for each of the six functional groups at each site by year.** Abbreviations are as follows, CSG = cool-season grasses (C3), Fescue = tall fescue (Schedonorus arundinaceus (Schreb.) Dumort., nom. cons.), Forbs = non-leguminous forbs, Legumes = leguminous forbs, Woody = woody vegetation, WSG = warm-season grasses (C4).

| Type | Site | Year | CSG | Fescue | Forbs | Legumes | Woody | WSG |
| --- | --- | --- | --- | --- | --- | --- | --- | --- |
| Graze-and-burn | Gilleland | 2007 | 28.32 | 59.52 | 17.68 | 7.88 | 1.30 | 0.11 |
|  |  | 2008 | 18.63 | 54.68 | 20.51 | 11.62 | 0.97 | 0.03 |
|  |  | 2009 | 24.92 | 60.17 | 28.37 | 20.86 | 0.46 | 0.17 |
|  |  | 2010 | 23.32 | 51.94 | 42.01 | 15.18 | 2.39 | 0.58 |
|  |  | 2011 | 19.42 | 56.81 | 51.07 | 21.80 | 9.86 | 4.72 |
|  |  | 2012 | 15.56 | 49.59 | 32.78 | 11.82 | 3.46 | 0.92 |
|  |  | 2013 | 22.06 | 54.06 | 25.11 | 10.46 | 6.97 | 0.66 |
|  |  | Average | 21.75 | 55.25 | 31.08 | 14.23 | 3.63 | 1.03 |
|  | Lee Trail | 2007 | 32.87 | 31.32 | 19.36 | 1.40 | 2.53 | 19.56 |
|  |  | 2008 | 15.20 | 30.74 | 17.08 | 6.13 | 2.69 | 8.16 |
|  |  | 2009 | 29.60 | 33.87 | 32.69 | 17.03 | 2.71 | 26.63 |
|  |  | 2010 | 17.24 | 26.89 | 32.19 | 12.87 | 1.69 | 31.23 |
|  |  | 2011 | 7.75 | 26.18 | 21.40 | 3.10 | 3.82 | 35.78 |
|  |  | 2012 | 19.50 | 24.19 | 21.04 | 4.19 | 2.22 | 25.56 |
|  |  | 2013 | 34.28 | 29.64 | 24.52 | 9.72 | 6.11 | 22.87 |
|  |  | Average | 22.35 | 28.98 | 24.04 | 7.78 | 3.11 | 24.25 |
|  | Pyland West | 2007 | 15.29 | 50.89 | 18.32 | 12.29 | 1.23 | 23.89 |
|  |  | 2008 | 8.88 | 36.84 | 23.99 | 25.24 | 1.41 | 9.97 |
|  |  | 2009 | 15.08 | 39.61 | 32.63 | 21.22 | 2.17 | 12.52 |
|  |  | 2010 | 9.72 | 38.28 | 36.63 | 29.74 | 2.03 | 11.08 |
|  |  | 2011 | 5.87 | 33.64 | 26.47 | 16.21 | 1.98 | 17.68 |
|  |  | 2012 | 32.01 | 33.93 | 52.68 | 15.28 | 1.17 | 19.21 |
|  |  | 2013 | 29.30 | 34.50 | 17.18 | 25.92 | 1.30 | 17.00 |
|  |  | Average | 16.59 | 38.24 | 29.70 | 20.84 | 1.61 | 15.91 |
| Patch-burn graze | Pyland North | 2007 | 15.35 | 50.34 | 16.16 | 12.12 | 0.92 | 21.28 |
|  |  | 2008 | 17.62 | 40.49 | 22.46 | 30.03 | 1.51 | 6.44 |
|  |  | 2009 | 23.21 | 44.78 | 22.72 | 31.82 | 2.14 | 9.57 |
|  |  | 2010 | 24.38 | 32.27 | 39.39 | 23.10 | 0.31 | 8.92 |
|  |  | 2011 | 12.09 | 38.61 | 23.42 | 15.49 | 0.54 | 19.86 |
|  |  | 2012 | 10.89 | 49.00 | 10.54 | 7.09 | 1.83 | 17.84 |
|  |  | 2013 | 25.67 | 38.61 | 12.48 | 28.19 | 2.29 | 12.06 |
|  |  | Average | 18.46 | 42.01 | 21.02 | 21.12 | 1.36 | 13.71 |
|  | Pyland South | 2007 | 23.20 | 47.83 | 22.58 | 10.10 | 1.85 | 16.72 |
|  |  | 2008 | 14.15 | 33.76 | 19.93 | 31.26 | 0.84 | 3.02 |
|  |  | 2009 | 20.08 | 36.73 | 33.81 | 38.20 | 0.53 | 7.66 |
|  |  | 2010 | 18.18 | 31.20 | 37.74 | 41.50 | 0.03 | 11.04 |
|  |  | 2011 | 6.72 | 33.22 | 35.49 | 14.58 | 1.83 | 12.88 |
|  |  | 2012 | 25.60 | 43.42 | 26.53 | 24.60 | 0.72 | 10.22 |
|  |  | 2013 | 36.21 | 39.77 | 27.61 | 32.18 | 1.97 | 16.88 |
|  |  | Average | 20.59 | 37.99 | 29.10 | 27.49 | 1.11 | 11.20 |
|  | Ringgold South | 2007 | 24.92 | 33.86 | 21.18 | 11.99 | 6.08 | 9.33 |
|  |  | 2008 | 14.09 | 25.27 | 24.30 | 31.06 | 2.87 | 7.66 |
|  |  | 2009 | 40.27 | 38.47 | 28.32 | 24.69 | 3.64 | 12.38 |
|  |  | 2010 | 16.06 | 22.93 | 31.57 | 35.66 | 1.12 | 24.56 |
|  |  | 2011 | 13.42 | 17.53 | 33.57 | 11.77 | 4.28 | 25.02 |
|  |  | 2012 | 17.38 | 30.71 | 18.32 | 11.99 | 4.11 | 24.97 |
|  |  | 2013 | 13.91 | 34.59 | 19.17 | 19.82 | 6.34 | 18.96 |
|  |  | Average | 20.01 | 29.05 | 25.20 | 21.00 | 4.06 | 17.55 |
| Reference | Pawnee | 2007 | 18.20 | 0.03 | 57.35 | 1.37 | 10.13 | 48.28 |
|  |  | 2008 | 14.94 | 0.00 | 60.30 | 3.52 | 3.72 | 35.02 |
|  |  | 2009 | 19.88 | 0.99 | 53.74 | 1.93 | 9.96 | 56.50 |
|  |  | 2010 | 4.80 | 1.19 | 55.97 | 12.36 | 6.47 | 42.22 |
|  |  | 2011 | 15.57 | 0.87 | 71.98 | 9.76 | 16.61 | 46.48 |
|  |  | 2012 | 9.18 | 0.52 | 34.26 | 3.47 | 10.03 | 43.32 |
|  |  | 2013 | 26.96 | 0.71 | 64.87 | 11.60 | 17.59 | 35.34 |
|  |  | Average | 15.65 | 0.62 | 56.92 | 6.29 | 10.64 | 43.88 |
|  | Ringgold North | 2007 | 7.03 | 0.00 | 36.87 | 0.00 | 5.60 | 64.07 |
|  |  | 2008 | 27.31 | 1.43 | 37.40 | 1.42 | 2.17 | 28.04 |
|  |  | 2009 | 36.91 | 0.00 | 42.42 | 12.69 | 2.42 | 47.41 |
|  |  | 2010 | 3.00 | 0.00 | 34.28 | 20.98 | 1.23 | 43.57 |
|  |  | 2011 | 39.04 | 0.00 | 62.79 | 10.90 | 9.54 | 48.65 |
|  |  | 2012 | 15.30 | 0.00 | 26.24 | 5.72 | 3.77 | 39.83 |
|  |  | 2013 | 13.98 | 0.00 | 34.61 | 3.66 | 4.84 | 54.19 |
|  |  | Average | 20.37 | 0.20 | 39.23 | 7.91 | 4.22 | 46.54 |
