# Supplementary material for: Exotic-Dominated Grasslands Show Signs of Recovery with Cattle Grazing and Fire
Source: PLoS One. 2016 Nov 7;11(11):e0165758. doi: 10.1371/journal.pone.0165758 (PMC5098731; doi:10.1371/journal.pone.0165758)
Supplement: S2 Table — Total abundance is the sum of all butterflies counted from six transects within each site in each year. (DOCX) [file pone.0165758.s002.docx]

**S2 Table: Total abundance of the 14 most common butterfly species for each site by year.** Total abundance is the sum of all butterflies counted from six transects within each site in each year.

| Type | Site | Year | All Butterflies | *Boloria bellona* | *Cercyonis pegala* | *Colias eurytheme* | *Colias philodice* | *Cupido comyntas* | *Danaus plexippus* | *Junonia coenia* | *Papilio polyxenes* | *Phyciodes tharos* | *Pieris rapae* | *Polites themistocles* | *Pyrisitia lisa* | *Speyeria cybele* | *Speyeria idalia* |
| --- | --- | --- | --- | --- | --- | --- | --- | --- | --- | --- | --- | --- | --- | --- | --- | --- | --- |
| Graze-and-burn | Gilliland | 2007 | 61 | 4 | 2 | 0 | 22 | 23 | 0 | 2 | 2 | 1 | 2 | 0 | 0 | 0 | 0 |
|  |  | 2008 | 90 | 0 | 4 | 2 | 2 | 46 | 2 | 7 | 0 | 4 | 10 | 2 | 0 | 1 | 0 |
|  |  | 2009 | 26 | 0 | 1 | 3 | 4 | 10 | 0 | 0 | 0 | 3 | 1 | 0 | 0 | 2 | 0 |
|  |  | 2010 | 126 | 2 | 13 | 20 | 7 | 27 | 0 | 12 | 9 | 24 | 0 | 6 | 0 | 1 | 0 |
|  |  | 2011 | 119 | 0 | 2 | 6 | 5 | 77 | 0 | 0 | 0 | 16 | 4 | 0 | 0 | 5 | 0 |
|  |  | 2012 | 77 | 0 | 4 | 6 | 1 | 35 | 0 | 8 | 0 | 12 | 4 | 0 | 0 | 4 | 1 |
|  |  | 2013 | 135 | 0 | 5 | 6 | 10 | 59 | 2 | 9 | 5 | 33 | 1 | 0 | 0 | 3 | 0 |
|  |  | Average | 90.57 | 0.86 | 4.43 | 6.14 | 7.29 | 39.57 | 0.57 | 5.43 | 2.29 | 13.29 | 3.14 | 1.14 | 0.00 | 2.29 | 0.14 |
|  | Lee Trail | 2007 | 13 | 0 | 0 | 0 | 4 | 3 | 2 | 0 | 0 | 0 | 0 | 0 | 0 | 1 | 1 |
|  |  | 2008 | 52 | 0 | 9 | 4 | 4 | 21 | 0 | 1 | 1 | 6 | 1 | 0 | 0 | 2 | 1 |
|  |  | 2009 | 12 | 1 | 1 | 0 | 0 | 0 | 0 | 0 | 0 | 4 | 1 | 1 | 0 | 1 | 1 |
|  |  | 2010 | 38 | 4 | 2 | 7 | 6 | 2 | 1 | 0 | 0 | 3 | 4 | 1 | 0 | 5 | 2 |
|  |  | 2011 | 45 | 0 | 8 | 0 | 0 | 9 | 1 | 0 | 1 | 18 | 1 | 0 | 0 | 1 | 5 |
|  |  | 2012 | 23 | 0 | 1 | 1 | 0 | 1 | 0 | 1 | 1 | 5 | 2 | 0 | 3 | 3 | 3 |
|  |  | 2013 | 66 | 0 | 1 | 5 | 3 | 23 | 1 | 3 | 0 | 22 | 1 | 1 | 2 | 2 | 0 |
|  |  | Average | 35.57 | 0.71 | 3.14 | 2.43 | 2.43 | 8.43 | 0.71 | 0.71 | 0.43 | 8.29 | 1.43 | 0.43 | 0.71 | 2.14 | 1.86 |
|  | Pyland West | 2007 | 62 | 8 | 1 | 0 | 15 | 10 | 0 | 2 | 3 | 13 | 10 | 0 | 0 | 0 | 0 |
|  |  | 2008 | 44 | 0 | 0 | 0 | 3 | 30 | 0 | 0 | 5 | 0 | 1 | 1 | 0 | 2 | 1 |
|  |  | 2009 | 19 | 1 | 0 | 4 | 2 | 2 | 3 | 0 | 1 | 2 | 1 | 0 | 0 | 1 | 2 |
|  |  | 2010 | 54 | 1 | 4 | 5 | 9 | 11 | 0 | 0 | 3 | 15 | 0 | 0 | 0 | 4 | 1 |
|  |  | 2011 | 53 | 2 | 5 | 1 | 4 | 23 | 0 | 0 | 0 | 7 | 2 | 1 | 0 | 5 | 0 |
|  |  | 2012 | 51 | 0 | 0 | 9 | 2 | 26 | 0 | 3 | 0 | 7 | 0 | 0 | 0 | 3 | 0 |
|  |  | 2013 | 158 | 0 | 3 | 6 | 12 | 60 | 0 | 4 | 0 | 63 | 2 | 3 | 0 | 3 | 0 |
|  |  | Average | 63.00 | 1.71 | 1.86 | 3.57 | 6.71 | 23.14 | 0.43 | 1.29 | 1.71 | 15.29 | 2.29 | 0.71 | 0.00 | 2.57 | 0.57 |
| Patch-burn graze | Pyland North | 2007 | 25 | 4 | 0 | 1 | 5 | 5 | 0 | 0 | 4 | 3 | 0 | 2 | 0 | 0 | 0 |
|  |  | 2008 | 50 | 0 | 1 | 2 | 2 | 27 | 0 | 0 | 5 | 8 | 0 | 1 | 0 | 0 | 1 |
|  |  | 2009 | 32 | 0 | 0 | 1 | 0 | 2 | 0 | 0 | 0 | 17 | 0 | 7 | 0 | 0 | 0 |
|  |  | 2010 | 34 | 1 | 1 | 5 | 10 | 5 | 0 | 1 | 2 | 6 | 0 | 1 | 0 | 0 | 0 |
|  |  | 2011 | 52 | 2 | 3 | 2 | 1 | 12 | 0 | 1 | 0 | 18 | 4 | 0 | 0 | 7 | 0 |
|  |  | 2012 | 74 | 1 | 0 | 6 | 1 | 48 | 2 | 0 | 0 | 7 | 2 | 1 | 0 | 5 | 0 |
|  |  | 2013 | 137 | 1 | 3 | 7 | 6 | 93 | 0 | 3 | 1 | 18 | 0 | 1 | 0 | 1 | 2 |
|  |  | Average | 57.71 | 1.29 | 1.14 | 3.43 | 3.57 | 27.43 | 0.29 | 0.71 | 1.71 | 11.00 | 0.86 | 1.86 | 0.00 | 1.86 | 0.43 |
|  | Pyland South | 2007 | 49 | 8 | 0 | 1 | 9 | 14 | 1 | 0 | 1 | 8 | 2 | 3 | 0 | 0 | 0 |
|  |  | 2008 | 73 | 0 | 2 | 2 | 3 | 52 | 0 | 3 | 4 | 5 | 1 | 0 | 0 | 0 | 1 |
|  |  | 2009 | 19 | 0 | 0 | 2 | 1 | 4 | 1 | 0 | 0 | 6 | 1 | 0 | 0 | 1 | 0 |
|  |  | 2010 | 87 | 6 | 2 | 10 | 5 | 10 | 1 | 0 | 1 | 48 | 0 | 0 | 0 | 2 | 0 |
|  |  | 2011 | 62 | 0 | 5 | 0 | 3 | 17 | 2 | 0 | 0 | 20 | 2 | 0 | 0 | 6 | 2 |
|  |  | 2012 | 63 | 1 | 0 | 3 | 2 | 31 | 0 | 0 | 0 | 20 | 0 | 0 | 3 | 2 | 0 |
|  |  | 2013 | 151 | 0 | 1 | 4 | 1 | 81 | 1 | 3 | 4 | 50 | 1 | 3 | 0 | 1 | 0 |
|  |  | Average | 72.00 | 2.14 | 1.43 | 3.14 | 3.43 | 29.86 | 0.86 | 0.86 | 1.43 | 22.43 | 1.00 | 0.86 | 0.43 | 1.71 | 0.43 |
|  | Ringgold South | 2007 | 36 | 5 | 0 | 3 | 7 | 12 | 1 | 0 | 1 | 6 | 0 | 0 | 0 | 0 | 1 |
|  |  | 2008 | 81 | 0 | 0 | 3 | 8 | 52 | 0 | 0 | 2 | 4 | 7 | 2 | 0 | 0 | 0 |
|  |  | 2009 | 18 | 0 | 0 | 1 | 3 | 4 | 0 | 0 | 0 | 7 | 0 | 0 | 0 | 1 | 1 |
|  |  | 2010 | 56 | 2 | 2 | 10 | 4 | 11 | 1 | 3 | 0 | 13 | 1 | 0 | 4 | 0 | 2 |
|  |  | 2011 | 58 | 1 | 1 | 0 | 0 | 41 | 0 | 0 | 0 | 9 | 0 | 0 | 0 | 2 | 1 |
|  |  | 2012 | 82 | 0 | 0 | 9 | 5 | 44 | 0 | 0 | 0 | 15 | 2 | 0 | 2 | 2 | 0 |
|  |  | 2013 | 116 | 1 | 0 | 3 | 1 | 51 | 0 | 5 | 1 | 40 | 2 | 0 | 0 | 5 | 7 |
|  |  | Average | 63.86 | 1.29 | 0.43 | 4.14 | 4.00 | 30.71 | 0.29 | 1.14 | 0.57 | 13.43 | 1.71 | 0.29 | 0.86 | 1.43 | 1.71 |
| Reference | Pawnee | 2007 | 103 | 1 | 17 | 0 | 2 | 3 | 1 | 0 | 0 | 6 | 1 | 0 | 0 | 2 | 68 |
|  |  | 2008 | 68 | 2 | 11 | 1 | 3 | 11 | 1 | 1 | 0 | 3 | 1 | 2 | 0 | 7 | 19 |
|  |  | 2009 | 58 | 1 | 10 | 0 | 0 | 3 | 2 | 0 | 0 | 15 | 1 | 0 | 0 | 1 | 25 |
|  |  | 2010 | 129 | 0 | 39 | 6 | 3 | 13 | 1 | 4 | 0 | 19 | 0 | 0 | 4 | 0 | 27 |
|  |  | 2011 | 111 | 3 | 32 | 2 | 3 | 9 | 1 | 0 | 0 | 9 | 2 | 0 | 0 | 11 | 36 |
|  |  | 2012 | 62 | 2 | 8 | 3 | 0 | 4 | 0 | 1 | 0 | 3 | 0 | 0 | 2 | 10 | 27 |
|  |  | 2013 | 91 | 0 | 8 | 2 | 3 | 32 | 0 | 4 | 0 | 28 | 0 | 1 | 0 | 1 | 8 |
|  |  | Average | 88.86 | 1.29 | 17.86 | 2.00 | 2.00 | 10.71 | 0.86 | 1.43 | 0.00 | 11.86 | 0.71 | 0.43 | 0.86 | 4.57 | 30.00 |
|  | Ringgold North | 2007 | 21 | 5 | 1 | 0 | 3 | 0 | 0 | 0 | 0 | 7 | 0 | 0 | 0 | 0 | 4 |
|  |  | 2008 | 27 | 0 | 3 | 2 | 2 | 3 | 3 | 0 | 1 | 2 | 0 | 1 | 0 | 0 | 9 |
|  |  | 2009 | 42 | 0 | 11 | 0 | 0 | 1 | 5 | 0 | 0 | 10 | 1 | 1 | 0 | 3 | 8 |
|  |  | 2010 | 50 | 4 | 9 | 2 | 7 | 2 | 2 | 1 | 0 | 0 | 4 | 0 | 0 | 8 | 10 |
|  |  | 2011 | 27 | 1 | 3 | 0 | 0 | 2 | 0 | 0 | 0 | 5 | 0 | 0 | 0 | 5 | 7 |
|  |  | 2012 | 47 | 0 | 3 | 2 | 1 | 2 | 0 | 4 | 0 | 5 | 0 | 0 | 18 | 1 | 7 |
|  |  | 2013 | 86 | 0 | 4 | 3 | 4 | 8 | 5 | 9 | 2 | 31 | 0 | 1 | 1 | 4 | 11 |
|  |  | Average | 42.86 | 1.43 | 4.86 | 1.29 | 2.43 | 2.57 | 2.14 | 2.00 | 0.43 | 8.57 | 0.71 | 0.43 | 2.71 | 3.00 | 8.00 |
